# Idiopathic Spinal Epidural Lipomatosis: Unusual Presentation and Difficult Management

**DOI:** 10.1155/2021/4562312

**Published:** 2021-02-12

**Authors:** Ouidad Louachama, Noureddine Rada, Ghizlane Draiss, Mohamed Bouskraoui

**Affiliations:** Mother and Child Hospital, Mohamed VI Marrakesh University Hospital, Marrakesh, Morocco

## Abstract

Epidural lipomatosis (EL) is a pathology characterized by abnormal accumulation of unencapsulated fat in the epidural space. Although rare, it is a possible cause of lumbosciatica or narrow lumbar canal in adults. It is often associated with favorable factors such as prolonged corticosteroid therapy or obesity. We report an observation of an 18-month-old child who presented with walking delay without other abnormalities, and the radiological exploration confirmed the lumbar epidural lipomatosis. The management was mainly symptomatic, based on motor physiotherapy with additional management in neurosurgery. Various etiologies can cause this disease, remain rare in pediatrics, and the idiopathic form is predominant in children.

## 1. Introduction

Epidural lipomatosis is a rare disease entity, and it is a pathologic accumulation of fat in the epidural space. Although EL may be asymptomatic, patients often have symptoms related to nerve or spinal cord compression [1].

## 2. Case Report

The case was an 18-month-old child, the youngest of the two siblings; his parents were not consanguineous, and had no history of maternal drug intake, hypothyroidism, or other diseases; the child was born by vaginal delivery, in term without perinatal asphyxia, and the birth weight was 3200 g.

The patient's psychomotor development was normal until the age of one year; he crawled and can stand with assistance, but he presented a gait delay without other associated signs especially no amyotrophy or orthopedic deformations.

General physical examination was normal with no evidence of facial dysmorphism, pulse rate of 85, and respiratory rate of 23; his growth was normal with a weight of 12 kg (M), height of 81 cm (+1 standard deviation), and head circumference of 46 cm (−1 standard deviation).

Neurological examination showed a reactive patient, standing with support; deep tendon reflexes were decreased, the plantar reflex was indifferent to both feet, tone was normal, and two blue spots of 1 cm were seen on the seat and foot.

The assessment consisted of an electromyography (EMG): without anomalies, the thyroid-stimulating hormone (TSH) level: normal (2,421 *μ*UI/ml and T4: 13.26 pmol/l); and creatine phosphokinase (CPK): normal (109 U/l). It is not in favor of a myopathy.

So, the flaccid pyramidal syndrome was suspected, and a cerebral spinal magnetic resonance imaging (MRI) revealed infiltration fat of distal lumbar epidural reducing the scabbard and dural bag, and the level of the medullary cone is located in L1-L2 in favor of epidural lipomatosis.

Searching for other associated neurological abnormalities, the urodynamic assessment was normal and the etiological investigation showed a normal rate of triglycerides (0.36 g/l) and total cholesterol (1.4 g/l).

The patient management consisted of motor physiotherapy with regular follow-up in consultation of neurosurgery, with a stationary evolution, and currently, walking is not yet acquired.

## 3. Discussion

Epidural lipomatosis (EL) is a rare condition characterized by the accumulation of unencapsulated fat within the epidural space. This can lead to a direct mass effect on the spinal cord or compression from venous engorgement through the compacted anterior and posterior external venous plexuses [2].

EL was first described by Lee and colleagues [3] in an adolescent who was receiving treatment with exogenous steroids after a kidney transplant. Most cases of EL are associated with long-term use of exogenous steroids, but a number of cases have been reported, which are associated with Cushing's disease, hypothyroidism, and pituitary prolactinoma [4].

In the literature for adults with epidural lipomatosis, the use of exogenous steroids is present in more than half of the cases reported, while obesity is present in around a quarter [2]. However, in a minority of cases, the cause of EL is idiopathic [5].

In our case, the drug intake, hypothyroidism, and obesity were not observed in the mother nor the child.

The patients with symptomatic EL may have radiculopathy, myelopathy, claudication, cauda equina syndrome, or paraplegia. These symptoms are most likely caused by compression due to excess fatty tissue in the epidural space, and the exact presentation depends on the location and degree of compression [1].

So, cervical involvement is rare. Symptoms related to epidural lipomatosis are thought to result from compression of the spinal cord and nerve root [6].

But in our case, the walking delay is the only inaugural symptom without peripheral neurological cause or myopathy, and no urodynamic disorders were observed.

To date, no definitive treatment has been reported for EL. Management of epidural lipomatosis can be conservative treatment or surgery. Conservative therapies include medication, weight loss, and activity modification. Nonsteroidal anti-inflammatory drugs (NSAIDs) and opioids can be administered to these patients. Surgical decompression is performed when paraparesis, urinary retention, or neurological deficit is severe or if the patient cannot stop the steroids [7].

About 90% of cases end up being treated surgically, but in patients with secondary disease in the lumbar region, this number appears to be closer to 65% [8].

This suggests that the location of the lesion should be considered when planning treatment. There may also be a correlation between the severity of the clinical presentation and the delay. The underlying etiology of EL should also guide therapeutic decisions [1].

So, in our observation, the initial treatment was conservative because we are not sure that EL would cause walking delay and may be just incidental finding, and no surgical management is decided considering its stationary evolution and its normal urodynamic assessment.

## 4. Conclusion

Epidural lipomatosis is a rare disease in pediatrics. MRI facilitated the diagnosis, but through this case report, we highlight the clinical presentation and the therapeutic difficulties noted in this population.

## Data Availability

The data used to support the findings of this study are included within the article.
